# IL-8 and its role as a potential biomarker of resistance to anti-angiogenic agents and immune checkpoint inhibitors in metastatic renal cell carcinoma

**DOI:** 10.3389/fonc.2022.990568

**Published:** 2022-08-19

**Authors:** Mimma Rizzo, Luca Varnier, Gaetano Pezzicoli, Marta Pirovano, Laura Cosmai, Camillo Porta

**Affiliations:** ^1^ Division of Medical Oncology, Azienda Ospedaliero Universitaria Consorziale Policlinico di Bari, Bari, Italy; ^2^ Department of Pediatrics, Meyer’ Childrens University Hospital, Florence, Italy; ^3^ Department of Interdisciplinary Medicine, School of Medicine, University of Bari “A. Moro”, Bari, Italy; ^4^ Division of Nephrology and Dialysis, Azienda Socio-Sanitaria Territoriale (ASST) Fatebenefratelli-Sacco, Fatebenefratelli Hospital, Milan, Italy; ^5^ Chair of Oncology, Interdisciplinary Department of Medicine, University of Bari “A. Moro”, Bari, Italy

**Keywords:** IL-8, biomarker of resistance, anti-angiogenic agent, immune checkpoint inhibitors, kidney cancer

## Abstract

The therapeutic armamentarium of metastatic Renal Cell Carcinoma (mRCC) has consistently expanded in recent years, with the introduction of VEGF/VEGFR (Vascular Endothelial Growth Factor/Vascular Endothelial Growth Factor Receptor) inhibitors, mTOR (mammalian Target Of Rapamycin) inhibitors and Immune Checkpoint (IC) inhibitors. Currently, for the first-tline treatment of mRCC it is possible to choose between a VEGFR-TKI (VEGFR-Tyrosine Kinase Inhibitor) monotherapy, an ICI-ICI (Immune Checkpoint Inhibitor) combination and an ICI-VEGFRTKI combination. However, a consistent part of patients does not derive benefit from first-line therapy with ICIs; moreover, the use of combination regimens exposes patients to significant toxicities. Therefore, there is a critical need to develop prognostic and predictive biomarkers of response to VEGFR-TKIs and ICIs, and measurement of serum IL-8 is emerging as a potential candidate in this field. Recent retrospective analyses of large phase II and phase III trials found that elevated baseline serum IL-8 correlated with higher levels of tumor and circulating immunosuppressive myeloid cells, decreased T cell activation and poor response to treatment. These findings must be confirmed in prospective clinical trials; however, they provide evidence for a potential use of serum IL-8 as biomarker of resistance to VEGFR-TKIs and ICIs. Considering the amount of new agents and treatment regimens which are transforming the management of metastatic renal cell carcinoma, serum IL-8 could become a precious resource in tailoring the best therapy for each individual patient with the disease.

## Introduction

Renal cell carcinoma (RCC) accounts for 80% of cases of kidney cancer, and it represents a major cause of morbidity and mortality worldwide (1). Up to 30% of RCC patients present with metastatic disease at diagnosis, and a similar percentage of patients with localized disease successfully removed through surgery will develop subsequent metachronous metastasis (2).

Clear cell renal cell carcinoma (ccRCC) is the most common histologic subtype, making up about 70% of cases of RCC (3), and for such reason, it has been extensively studied from a molecular point of view. The vast majority of cases of ccRCC presents with loss of function of the Von Hippel Lindau (VHL) gene (4), leading to unrestrained Hypoxia-Inducible Factor (HIF)-1α and HIF-2α activity and consequent enhanced cell growth and angiogenesis (5). However, VHL loss alone is not sufficient to induce tumor formation (3, 6). Several genes involved in chromatin remodeling (PBRM1, KDM5C, UTX, JARID1CM. SETD2), as well as genes involved in the PI3K-AKT-mTOR axis were proven to be mutated in RCC (7). Alterations in the PI3K-AKT-mTOR pathway increase tumor cell growth and proliferation as well as induce a metabolic rewiring in cancer cells (8). The high prevalence of these mutations underlies the current view on RCC ontogeny involving inactivation of pVHL as initiating step, followed by additional mutations in the aforementioned genes as subsequent events in tumor formation (4). The elucidation of such biological mechanisms has translated into clinical practice through the introduction of tyrosine kinase inhibitors, directed against VEGFR and similar proteins, and mTOR inhibitors, shutting down the mTOR complex 1 (mTORC1).

Before the introduction of such agents, first-line therapies for RCC relied upon the highly immunogenic nature of the tumor, which could be targeted with the use of high-dose interleukins and interferon (IFN)-alpha (9). This form of treatment generally had really poor response rates and survival benefits, but the presence of a small group of long-term responders underpinned the potentiality of immunotherapy in RCC. As immune checkpoint inhibitors (ICIs) revolutionized the management of several different cancers, their use has become a standard of care in advanced renal cell carcinoma too (1). The double ICI combination ipilimumab plus nivolumab has been approved as first-line treatment in IMDC intermediate and poor-risk patients (1, 10, 11). Several clinical trials tested ICIs in combination with VEGFR-TKIs, and results so far are showing unprecedented response rates and survival benefits across all patients’ risk groups (12, 13). As a result, the FDA has approved three combinations of an ICI and a VEGFR-TKI (pembrolizumab and axitinib, pembrolizumab and lenvatinib, nivolumab and cabozantinib) as first-line therapy across all patients’ risk groups (14). As the treatments are quickly expanding, physicians are facing new challenges in determining which therapeutic regimens is the most suitable for patients. The two major risk stratification models, the MSKCC and IMDC, are becoming obsolete as the new treatment regimens confer clinical benefits across all risk groups (12, 13). A great amount of work has focused on determining whether tumour and/or tumour-infiltrating immune cell protein expression of programmed cell death ligand 1 (PD-L1) could predict response to ICI therapy (11, 13, 15–17), but the results were controversial (18, 19). Similarly, other deeply-investigated biomarkers, such as CD8+ T cell density (13, 20, 21), tumor mutational burden (TMB) (21–23), PBRM1 mutation (21, 23, 24), have failed to yield uniform predictive results.

Ideally, biomarkers should be assessed in a minimally-invasive manner. In this respect measurement of serum IL-8 might represent a novel prognostic and predictive parameter in immunotherapy. Serum IL-8 has been recently analyzed in several different ICI trials for different cancers, including mRCC, and results suggest that IL-8 might represent a negative prognostic biomarker for solid tumors, but that it might also represent a biomarker of resistance to ICI treatment, hence aiding in predicting response to therapy (25, 26). In this review, we report the physiologic role of IL-8, its involvement in the process of carcinogenesis, its initial assessment as a clinical biomarker in cancer, and how these recent analyses of IL-8 in clinical trials may pave the way for a more thorough investigation of IL-8 as a prognostic and predictive biomarker of response to ICI and/or TKI therapy in mRCC.

## Physiology of IL-8

CXCL8, also known as interleukin (IL)-8, is one of the most extensively studied chemokines. It was first described in the late 1980s, where it was initially called neutrophil activating factor (NAF) due to its role in neutrophil exocytosis and oxidative burst (27, 28). IL-8 is a 6-8 KDa protein secreted by different cell types including blood monocytes, alveolar macrophages, fibroblasts, endothelial cells, and epithelial cells (29, 30). IL-8 expression is induced by various cytokines (IL-1, IL-6, CXCL12, TNF-α), hypoxic states, reactive oxygen species (ROS), bacterial particles, and other environmental stresses (29). Through the binding with its two receptors, CXCR1 and CXCR2, IL-8 exerts its major physiologic functions: promoting a pro-inflammatory state and stimulating angiogenesis. IL-8 is a potent chemoattractant molecule that drives mainly neutrophils but also monocytes to the site of inflammation (31, 32). Moreover, IL-8 favors the resolution of infections by acting mostly on neutrophils and promoting neutrophils-mediated phagocytosis, oxidative bursts, and release of neutrophil extracellular traps (32). In addition to its pro-inflammatory function, IL-8 acts to favor angiogenesis by promoting endothelial cells proliferation, survival, and migration, culminating in the formation of new blood vessels. This pro-angiogenic property favors the process of tissue healing from the inflammatory state (32).

## IL-8 and its role in carcinogenesis

IL-8 has been extensively explored in cancer research. Tumor cells shape the surrounding microenvironment through the expression and release of cytokines and chemokines. The IL-8/IL-8R axis plays an important role in such context; tumor cell acquisition of CXCR1 and CXCR2 and/or IL-8 is known to be a common event during tumor progression (29, 32), and, similarly, IL-8 and its receptors are widely expressed by a variety of non-malignant cells present in the tumor microenvironment, including tumor-associated macrophages, neutrophils and endothelial cells (33). The pro-tumorigenic effect of IL-8 within the TME is exerted *via* both autocrine and paracrine ways. Autocrine loops form on the surface of tumor cells, which concomitantly produce IL-8 and express its receptors. IL-8 signaling stimulates tumor growth by enhancing tumor cell growth (33, 34). Moreover, IL-8 signaling is emerging as an important factor in tumor cell survival, by promoting the expression of anti-apoptotic genes, particularly in the context of environmental (e.g. hypoxia) or treatment-induced stresses (35, 36). IL-8 has been directly involved in the process of epithelial-mesenchymal transition (EMT), where acquisition of a mesenchymal phenotype enhances tumor cell aggressiveness and invasion capacity, hence favoring metastasis (37).

**Figure 1:**
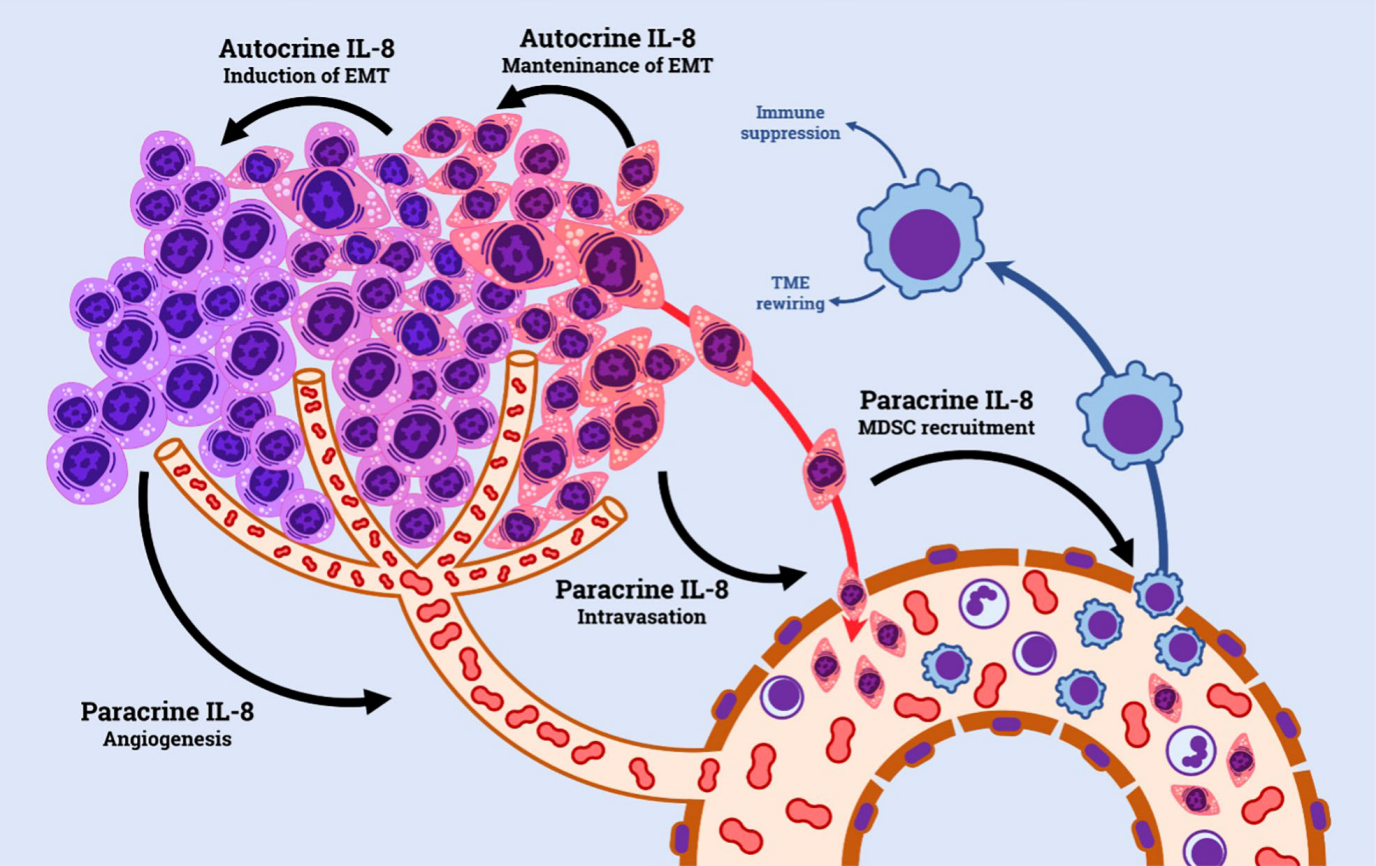
Principal mechanisms involved in IL-8-induced resistance to ICI and anti-angiogenic TKI.

In addition, by acting in a paracrine manner, IL-8/IL-8R axis has a prominent role in promoting a favorable tumor microenvironment by recruiting immune cells characterized by permissive phenotype for tumor growth, such as N2-neutrophils and myeloid-derived suppressor cells (MDSCs) (32). The presence of such cells in cancer has been associated with a more defective anti-tumor immune response within the TME, particularly by inhibiting T-cells (38, 39). (**Figure 1**)

Finally, high levels of IL-8 in the TME are potent stimulants for tumor angiogenesis. This is achieved through multiple mechanisms, including directly promoting endothelial cell proliferation and survival, up-regulating VEGF-A and its receptor VEGFR2, and inducing expression of matrix metalloproteases, which are capable of mobilizing sequestered pro-angiogenic factors (40, 41).

Overall, IL-8 pathways play an active part in promoting carcinogenesis, representing a potential therapeutic target in clinical setting (25, 42).

## Analysis of IL-8 as a biomarker in cancer

Measurement of serum IL-8, due to its short half-life, may represent a good candidate to accurately estimate the number of tumor cells producing this chemokine at any given time (43). These values might be exploited both pre-treatment, to estimate tumor burden, and on treatment, where changes in serum concentration could then reflect variations in tumor burden or tumor composition as well.

High IL-8 concentrations have been detected in serum and tissue specimens from patients with different cancers, and have been shown to correlate with a worse clinical stage of tumors (26, 43, 44), a more prominent tumor burden (45), presence of metastasis, and worse overall survival (44, 46). Vlachostergios et al. demonstrated that IL-8 levels, at baseline and after LPS-stimulation, are independent predictors of both PFS and OS in non-small-cell lung cancer (47).

In renal cell carcinoma, elevated serum IL-8 was associated with higher tumor burden and worse overall survival (43). Several studies also associated IL-8 up-regulation in cancers with resistance to chemotherapy (35, 48, 49) and targeted therapy (50, 51).

## IL-8 and resistance to immune checkpoint inhibitors

Evaluation of IL-8 in the context of immunotherapy has gained interest recently. Analysis of a small sample of patients with advanced melanoma and NSCLC treated with anti-PD1 monoclonal antibodies has shown that changes in serum IL-8 were associated with response to treatment (52). Of note, early changes in serum IL-8 levels, measured only 2-3 weeks after starting therapy, could predict response to treatment and overall survival, with patients witnessing a drop in IL-8 levels having better clinical outcomes compared to patients experiencing rising IL-8 levels (52). In this study, only changes in serum IL-8 were associated with significant clinical response, thereby strengthening the potential role of IL-8 as ICI biomarker (52). (**Table 1**)

**Table 1:** Principal retrospective data in literature exploiting the role of IL-8 as a predictor of resistance to ICI and anti-angiogenic TKI.

Reference	Study population	Study design	Outcome
Schalper et al. (52)	Retrospective analysis of 392 mRCC patients of the Checkmate-025 trial	Nivolumab vs Everolimus in mRCC patients progressed beyond ≥1 anti-angiogenic therapy	Longer OS (HR 2.56) and PFS (HR 1.36) in patients with low (<23 pg/ml) baseline serum IL-8 (p < 0.0001) in the Nivolumab arm. Longer OS (HR 2.40) in patients with low IL-8 (p< 0.0001) in the Everolimus arm.
Yuen et al. (53)	Retrospective analysis of 915 mRCC patients of the IMmotion 150 trial	Atezolizumab vs Atezolizumab + Bevacizumab vs Sunitinib in mRCC treatment-naïve patients	Longer OS (HR 2.55) in patients with low baseline serum IL-8 (p =0.017) in the Atezolizumab arm. Trend toward longer OS in patients with low baseline serum IL-8 the Atezolizumab à Bevacizumab and Sunitinib arm.
Tran et al. (54)	Retrospective analysis of 344 mRCC patients in the Pazopanib phase III trial	Pazopanib vs placebo in mRCC treatment-naïve and cytokine-pretreated patients	Longer PFS in patients with high concentrations (relative to median) of interleukin 8 (p=0·006) in the Pazopanib arm.
Iacovelli et al. (55)	Retrospective analysis of 36 patients with mRCC treated with targeted agents	mRCC patients treated with either Sunitinib, Pazopanib, Sorafenib, Bevacizumab, Temsirolimus	Higher 12 months-PFS in patients without immunohistochemical espression of interleukin 8 (p=0.009)
Sepe et al. (56)	Prospective analysis of 25 patients treated with Pazopanib	mRCC patients treated with Pazopanib as a first-line therapy	Low levels of baseline interleukin 8 associated with higher OR rate (p=0.047) and longer OS (p=0.04).

The role of baseline and on-treatment serum IL-8 has been evaluated in two major retrospective analyses of large phase II and phase III trials. The trials spanned several different cancers (53, 57), but a focus on the results related to the RCC trials is presented here. Schaper et al. analyzed the checkmate 025 trial, where patients with advanced renal cell carcinoma were randomized to either nivolumab or everolimus 16; using overall survival to obtain 23 pg/mL as clinically relevant stratification cut-off for serum IL-8 concentration, patients were independently stratified and analyzed in terms of progression-free survival (PFS), overall survival (OS) and objective response rate (ORR) (53). Results showed that in the group of patients treated with the ICI nivolumab, an OS hazard ratio of 2.56 (95% confidence interval 1.07-1.72, P < 0.0001) between patients with high (>23 pg/ml) and low (<23 pg/ml) baseline serum IL-8 was found. Similarly, the PFS (1.36, 95% CI 1.89-3.45, P <0.0001) was worse in patients with higher serum IL-8 levels; moreover, nivolumab-treated patients with serum IL-8 < 23 pg/mL had an ORR of 27.9%, as opposed to ORR of 19.5% in patients with serum IL-8>23 pg/mL. Interestingly, the association between elevated baseline serum IL-8 level and reduced survival was also observed in everolimus arm (HR=2.40, 95% CI 1.78-3.22, P < 0.0001). These results were consistent across treatment and tumor types, supporting the view of IL-8 as a global biomarker of poor prognosis in cancer. In addition, serum IL-8 was positively correlated with tumor IL-8 gene expression. High tumoral IL-8 was associated with tumoral infiltration of specific subsets of inflammatory cells, including neutrophils and monocytes, while and IFN-γ and T-cell transcripts signatures were downregulated. These findings point towards a link between IL-8 and an immunosuppressive tumor microenvironment highly infiltrated by myeloid cells with decreased antitumoral adaptive T-cell response.

The analysis performed by Yuen et al. focused on the possible correlation between plasma, peripheral blood mononuclear cell (PBMCs), and intratumoral IL-8 and clinical outcomes in the phase II IMmotion 150 trial, where patients with treatment-naïve mRCC were randomized to atezolizumab monotherapy or atezolizumab plus bevacizumab versus sunitinib (57). Elevated plasma IL-8 was associated with worse OS in the atezolizumab monotherapy arm (HR, 2.55, 95% CI 1.18-2.55, P = 0.017), while a trend towards worse OS in the atezolizumab + bevacizumab (HR, 1.25, 95% CI 0.61-2.60, P = 0.535) and sunitinib (HR, 1.48; 95% CI 0.69-3.20, P = 0.314) was observed (57). Using single-cell RNA sequencing, IL-8 expression was shown to be more prominent in the peripheral mononuclear myeloid cluster compared to the mononuclear lymphoid cluster and, concomitantly, within individual myeloid subsets, including monocyte, dendritic cells, and DC-like clusters, increased IL-8 expression was associated with both enrichment of myeloid inflammatory genes and downregulation of genes associated with the antigen-presentation machinery, such as HLA genes and interferon-γ-induced genes. A similar gene signature was seen in myeloid cells infiltrating the tumor. This fact may underlie a defective anti-tumoral antigen presentation machinery in the presence of overexpressed IL-8. Elevated IL-8 gene expression in the tumor correlated with higher neutrophils within the tumor (53, 57),. Additionally, high tumor IL-8 gene expression was associated with worse OS in mRCC treated with atezolizumab monotherapy; importantly, high tumor IL-8 expression remained associated with worse OS even in T cell-infiltrated tumors in mRCC patients treated with atezolizumab (HR, 15.6; 95% CI, 3.15, 77.6; P = 0.0004), but not in the atezolizumab + bevacizumab group (HR, 0.96; 95% CI, 0.29, 3.2; P = 0.945) and sunitinib group (HR, 1.94; 95% CI, 0.67, 5.6; P = 0.225).

## IL-8 and resistance to anti-angiogenic TKI

The idea that the changes in tumor microenvironment induced by TKI could improve the efficacy of ICI has been suggested by many preclinical data (58). The results from recent clinical data seem to confirm this hypothesis (12, 59). Hence the tendency toward ICI plus TKI combinations. (**Table 1**)

IL-8 could be useful in this setting, since it could predict resistance to TKI (60). In 2010, Huang et al. observed that tumors developing alternative angiogenic pathways are often those with increased expression of tumor-derived IL-8. Up-regulation of IL-8 may thus activate proangiogenic pathways that may functionally compensate for the inhibition of VEGF-VEGFR-dependent angiogenesis (61). It has been documented that the hyper-expression of IL-8 leads to VEGF mRNA transcription and autocrine VEGFR-2 activation (62). Moreover, this cytokine can induce the epithelial-to-mesenchimal transition *via* AKT activation in RCC cells, thus rendering them more resistant to VEGFR inhibition (54).

Exploration of plasma IL-8 as a potential prognostic biomarker in patients treated with the anti-angiogenic agent pazopanib has been performed both retrospectively (55, 56) and prospectively in a small cohort of patients with mRCC (63). Similarly, a prospective study analyzed baseline serum IL-8 and clinical outcomes in patients with mRCC receiving sunitinib (64). Results obtained suggest a potential negative prognostic value for plasma IL-8, with elevated plasma concentration associated with worse clinical outcomes upon treatment with anti-angiogenic TKIs as compared to lower plasma concentration (55, 63)

## Discussion

Overall, this review highlights the potential role of IL-8 as a driver of resistance to immune checkpoint inhibitors. While the findings reported in the Checkmate-025 trial point towards a generalized role of IL-8 as a negative prognostic biomarker, both in ICIs and TKI regimens, Yuen et al. found that the effect of plasma IL-8 on clinical outcomes appeared to be more pronounced in single-agent ICI. These findings suggest that higher baseline IL-8 may be selectively predictive of which patients are less likely to benefit from ICI monotherapy. This point can be particularly relevant in the management of metastatic renal cell carcinoma, where continuously expanding therapeutic options calls for the rapid development of new biomarkers that could allow selection of the proper treatment regimen for each individual patient, thereby maximizing survival and concomitantly limiting toxic adverse effects. Besides its direct stimulation of cancer cell proliferation and promotion of angiogenesis, high tumoral IL-8 levels reflect a unique, unfavorable tumor microenvironment characterized by prominent myeloid-cell infiltration and suppression of adaptive T-cell anti-tumor response (53). High-tumoral IL-8 expression is associated with recruitment of several myeloid cells lines, including MDSCs, CD15+ monocytes, and neutrophils, which have all been demonstrated to impair adaptive T cell antitumor immunity by several different mechanisms (39, 65, 66). Transcriptomic characterization of circulating and tumor-infiltrating IL-8-producing MDSCs demonstrated an increased expression of myeloid pro-inflammatory genes and downregulation of antigen-presentation and interferon-inducible genes, underlying impairment of adaptive immunity (57).

This deleterious effect of MDSCs on anti-tumor adaptive immunity might directly affect resistance to immunotherapies in cancer (67). In the phase II IMmotion 150 trial, the authors conducted exploratory analyses of molecular biomarkers relevant to the disease and tumor immune biology in mRCC, and their potential association with clinical outcomes within each treatment group and across treatment groups (68). Distinct biological subgroups were obtained, based on the relative expression of angiogenesis, lymphocitic, and myeloid inflammation-associated genes. Atezolizumab monotherapy was effective on tumor with pre-existing immunity and a relatively lower expression of myeloid inflammation-associated genes (Teff-high/Myeloid-low), but less so in immunogenic tumors with concomitantly high myeloid inflammation (Teff-high/Myeloid-high) (21). These findings underscore the relevance of a myeloid inflammatory milieu in determining resistance to ICI, even in the presence of a strong T cell inflammatory response, which is normally associated with better outcomes with ICIs therapy (69).

Conversely, the combination atezolizumab plus bevacizumab showed improved PFS compared to atezolizumab monotherapy in the Teff-high/Myeloid-high biological subgroups (HR 0.25; 95% CI, 0.10-0.60). This is in line with previous findings delineating an immunosuppressive role for VEGF, on top of its pro-angiogenic function, by impairing dendritic cell maturation, T-cell function, and promoting the proliferation of MDSCs (70). Consequently, VEGF/VEGFR blockade is thought to exert an anti-tumor immunomodulatory effect and has been shown to reduce MDSCs in tumors and blood in both preclinical tumor models and human cancers (71). In the context of a highly inflamed TME with infiltration of both T cell and MDSCs (Teff-high/Myeloid-high), the addition of an anti-VEGF/VEGFR agent (bevacizumab) to an immune checkpoint inhibitor (atezolizumab) may overcome innate inflammation-mediated resistance in these tumors, and synergistically enhance the reinvigorating effects of ICI on adaptive antitumor immunity. As increased serum IL-8 levels correlate with bulk tumor IL-8 gene expression and with tumor and circulating MDSCs (66), measurement of its serum concentration might be indicative of the myeloid inflammatory state of tumors, and therefore whether ICI monotherapy could be effective or the addition of a VEGF/VEGFR inhibitor should be considered. In this regard, IL-8 could be used as a predictive marker of response to immunotherapy and might be part of a comprehensive biomarker signature, that could contribute to personalized therapy in patients with mRCC. The current findings provide a rationale for the use of IL-8 as a potential prognostic and predictive biomarker in the use of ICIs and TKI in mRCC, but a limitation of this conclusion relies on the retrospective nature of the results reported. To ensure that the relevance of such data translates soon into clinical practice, IL-8 must be evaluated in prospective biomarker clinical trials.

## Author contributions

MR, LV, and CP wrote sections of the manuscript. All authors contributed to manuscript revision, read and approved the submitted version.

## Conflict of interest

The authors declare that the research was conducted in the absence of any commercial or financial relationships that could be construed as a potential conflict of interest.

## Publisher’s note

All claims expressed in this article are solely those of the authors and do not necessarily represent those of their affiliated organizations, or those of the publisher, the editors and the reviewers. Any product that may be evaluated in this article, or claim that may be made by its manufacturer, is not guaranteed or endorsed by the publisher.
